# Acute Heart Failure After Reperfused Ischemic Stroke: Association With Systemic and Cardiac Inflammatory Responses

**DOI:** 10.3389/fphys.2021.782760

**Published:** 2021-12-21

**Authors:** Lilian Vornholz, Fabian Nienhaus, Michael Gliem, Christina Alter, Carina Henning, Alexander Lang, Hakima Ezzahoini, Georg Wolff, Lukas Clasen, Tienush Rassaf, Ulrich Flögel, Malte Kelm, Norbert Gerdes, Sebastian Jander, Florian Bönner

**Affiliations:** ^1^Division of Cardiology, Pulmonology, and Vascular Medicine, Medical Faculty, University Hospital Düsseldorf, Düsseldorf, Germany; ^2^Department of Neurology, Medical Faculty, University Hospital Düsseldorf, Düsseldorf, Germany; ^3^Experimental Cardiovascular Imaging, Department of Molecular Cardiology, Medical Faculty, Heinrich-Heine-University Düsseldorf, Düsseldorf, Germany; ^4^Department of Biology, Institute of Metabolic Physiology, Heinrich-Heine University, Düsseldorf, Germany; ^5^Department of Cardiology and Vascular Medicine, West German Heart and Vascular Center, Medical Faculty, University Hospital Essen, Essen, Germany; ^6^Cardiovascular Research Institute Düsseldorf (CARID), Heinrich Heine University, Düsseldorf, Germany

**Keywords:** stroke, tMCAO, heart failure, myocardial injury, inflammation, MRI, echocardiography

## Abstract

Patients with acute ischemic stroke (AIS) present an increased incidence of systemic inflammatory response syndrome and release of Troponin T coinciding with cardiac dysfunction. The nature of the cardiocirculatory alterations remains obscure as models to investigate systemic interferences of the brain-heart-axis following AIS are sparse. Thus, this study aims to investigate acute cardiocirculatory dysfunction and myocardial injury in mice after reperfused AIS. Ischemic stroke was induced in mice by transient right-sided middle cerebral artery occlusion (tMCAO). Cardiac effects were investigated by electrocardiograms, 3D-echocardiography, magnetic resonance imaging (MRI), invasive conductance catheter measurements, histology, flow-cytometry, and determination of high-sensitive Troponin T (hsTnT). Systemic hemodynamics were recorded and catecholamines and inflammatory markers in circulating blood and myocardial tissue were determined by immuno-assay and flow-cytometry. Twenty-four hours following tMCAO hsTnT was elevated 4-fold compared to controls and predicted long-term survival. In parallel, systolic left ventricular dysfunction occurred with impaired global longitudinal strain, lower blood pressure, reduced stroke volume, and severe bradycardia leading to reduced cardiac output. This was accompanied by a systemic inflammatory response characterized by granulocytosis, lymphopenia, and increased levels of serum-amyloid P and interleukin-6. Within myocardial tissue, MRI relaxometry indicated expansion of extracellular space, most likely due to inflammatory edema and a reduced fluid volume. Accordingly, we found an increased abundance of granulocytes, apoptotic cells, and upregulation of pro-inflammatory cytokines within myocardial tissue following tMCAO. Therefore, reperfused ischemic stroke leads to specific cardiocirculatory alterations that are characterized by acute heart failure with reduced stroke volume, bradycardia, and changes in cardiac tissue and accompanied by systemic and local inflammatory responses.

## Introduction

Up to two-thirds of patients with acute ischemic stroke (AIS) present with electrocardiogram (ECG) abnormalities, cardiac contractile dysfunction, and increased levels of circulating high-sensitive Troponin T (hsTnT), indicative of myocardial infarction within the first 24 h after stroke (Scheitz et al., 2014; Mochmann et al., 2016; Krause et al., 2017; Chen et al., 2017b). In ~75% of patients with AIS and coinciding hsTnT elevation, no coronary culprit lesion is identified or even absence of any obstructive coronary artery disease can be documented (Zeus et al., 2016). Thus, the AIS-induced release of cardiac biomarkers in combination with ECG abnormalities and non-territorial contractile dysfunction does not fulfill the definition of Type 1 myocardial infarction (Ibanez et al., 2018). The entity of myocardial infarction with non-obstructive coronary arteries (MINOCA) mirrors at least some cardiac features observed after AIS (Agewall et al., 2017). Indeed, 19% of patients experience a fatal or non-fatal cardiac event within days after AIS (Chen et al., 2017b) and hsTnT elevation predicts mortality in patients after AIS (Scheitz et al., 2014). The exact nature of AIS-induced acute cardiac alterations remains unclear at present and treatment options are lacking.

Specific brain regions are implicated in autonomic activation after stroke (Smith et al., 1986) and the insular cortex is considered to play a central role in autonomic regulation (Soros and Hachinski, 2012). In patients with AIS, ECG irregularities and plasma hsTnT elevation are frequent when the insular cortex is involved (Krause et al., 2017). Injury to the right-sided insular cortex results in an increased rate of sudden cardiac death (Tokgozoglu et al., 1999). ECG findings in patients after stroke point to a parasympathetic effect on the heart (Christensen et al., 2005; Tahsili-Fahadan and Geocadin, 2017).

Several clinical and experimental findings indicate a neuro-inflammatory network that may play a critical role for the interaction between the ischemic brain and the heart (Ishikawa et al., 2013). Ischemic damage of the insular cortex, which is a major regulator of autonomic balance, can result in direct cardiac damage with myocytolysis due to exacerbated catecholamine action (Smith et al., 1986; Soros and Hachinski, 2012). Right sided insular lesions result in parasympathetic overdrive with considerably mortality (Christensen et al., 2005). AIS induces massive immune responses in the brain and the systemic immune compartment that correlate to the severity of initial brain damage (Audebert et al., 2004; Flogel et al., 2008). Markers of inflammation predict mortality after stroke, as shown for leukocytes (Furlan et al., 2014). C-reactive protein (CRP; Muir et al., 1999) and interleukin (IL)-6 (Pusch et al., 2015). Paradoxically, the subacute phase after stroke is characterized by a profound immunosuppression with splenic atrophy, lymphopenia, and a shift from pro-inflammatory T_H_1-type cytokines toward anti-inflammatory T_H_2 cytokines and expansion of the number of regulatory T cells (Prass et al., 2003; Offner et al., 2006). The mechanisms involved in this inflammatory switch are incompletely understood and a potential link between inflammation and cardiac injury has not been studied so far (Prass et al., 2003).

Experimental evidence in models of transient middle cerebral artery occlusion (tMCAO) for 30 min indicates long-term effects of the ischemic brain on the heart leading to chronic heart failure that is treatable by beta blockers (Bieber et al., 2017). However, the nature of AIS-induced acute heart failure at early time points has not been examined in detail and murine models to comprehensively study mechanisms of acute cardiac alterations following reperfused AIS are sparse in general (Min et al., 2009).

Accordingly, we established a model to systematically characterize the acute cardiac and circulatory response to a more robust ischemic stroke in mice. Thus, we used 60 min of ischemia with subsequent reperfusion to address the question whether experimental stroke induces specific patterns of cardiac and circulatory alterations leading to acute heart failure.

### Animals

All animal studies were approved by the Local Animal Agency (State Agency for Nature, Environment and Consumer Protection (LANUV), registration number: AZ 84-02-04-14.A338) and performed in accordance with the recommendations of the European Ethical Committee (EEC; 2010/63/EU) for the care and use of laboratory animals. Male, 12–14 weeks-old C57BL/6 J mice (Janvier, Le Genest-Saint-Isle, France) with a body weight between 24 and 30 g were used. All mice had access to regular chow and water *ad libitum* and were housed in a 12 h light/12 h dark cycle.

### Transient Middle Cerebral Artery Occlusion (tMCAO)

Mice were anesthetized using a mixture of 3% (v/v) isoflurane (Isofluran-Piramal®, Piramal Critical Care, Germany) in 40% oxygen in room air for induction and was reduced to 1.5% (v/v) isoflurane for maintain anesthesia. Before surgery mice were injected subcutaneously with 0.05 mg/kg buprenorphine (Temgesic®, Indivior, United Kingdom). During surgery, body temperature was controlled and maintained using a heating plate. For transient occlusion of the middle cerebral artery (MCA) a silicon-coated thread (6-0 medium MCAO suture L910 PK10, Doccol corporation, United States) was inserted *via* the right internal carotid artery and advanced into the MCA, left in place for 60 min, with subsequent reperfusion as described previously (Clark et al., 1997). Sham animals underwent the same procedure except for the insertion of the thread. To monitor heart rate during surgery an electrocardiogram (ECG Amplifier Type 689, Hugo Sachs, March, Germany) was recorded by LabChart software (LabChart Pro 7, adinstruments, Dunedin, New Zealand). All animals were operated by the same experimenter to minimize stroke size variability. Postoperatively and also 24 h after surgery, mice were assessed by a clinical neurological examination according to Bederson (Bederson et al., 1986; Supplementary Table T1). For quantification of stroke volume, animals were injected intraperitoneal with 10 mg/kg xylazine (Rompun®, 2%, Bayer, Leverkusen, Germany) and 100 mg/kg ketamine (Ketanest®, Pfizer, Berlin, Germany), killed *via* exsanguination 24 h post tMCAO after completion of the cardiac analysis (see below). Mice were decapitated, brains were removed, cut into 2 mm-thick coronal sections, and stained by 2% 2,3,5-triphenyltetrazolium chloride (TTC; Sigma-Aldrich, St. Louis, United States) for 5 min at 37°C. Planimetric calculation using ImageJ software (NIH, Bethesda, United States) was used to quantify the ischemic area relative to total brain volume (Min et al., 2009). In a subset of mice stroke volume was measured using magnetic resonance imaging (MRI) in T2-weighted multi-slice spin echo images.

### Measurements of Cardiac Function

#### Echocardiography

Left ventricular function as evaluated 24 h after tMCAO by transthoracic echocardiography using a MS-400 scanhead (Vevo 2100, VisualSonics, FUJIFILM, Toronto, Canada). For the echocardiographic characterization mice were anesthetized with 1.5% (v/v) isoflurane. Standard sections of the heart (long axis, short axis in B and M mode, mitral valve flow, aorta ascendens flow) were recorded as previously described (Erkens et al., 2015). Vevo lab software 2.2.0 (VisualSonics) was used for evaluation of ejection fraction (EF), stroke volume (SV), fractional shortening (FS), cardiac output (CO), end-systolic- and end-diastolic volumes (ESV; EDV), and parameters of cardiac deformation [global longitudinal strain (GLS), early diastolic strain rate (SRe), regional longitudinal and radial strain and strain rate].

#### Invasive Hemodynamics

Twenty four hours after tMCAO hemodynamics were measured in the aorta and left ventricle using a 1.4 F Mikro-Tip® Catheter (model SPR-839, Millar Instrument, Houston, United States) as described previously (Pacher et al., 2008; Erkens et al., 2015). Mice were kept under 1.5% (v/v) isoflurane anesthesia and constant body temperature during the procedure. Iox-2 software (EMKA technologies, Paris, France) recorded the following parameters: maximum (*P*_sys_), minimum (*P*_dias_), and mean (*P*_mean_) arterial pressure in the aorta as well as end-systolic and end-diastolic pressure (LVESP; LVEDP), maximum and minimum delta pressure/delta time (dp/dt) in the left ventricle.

#### Magnetic Resonance Imaging

For cardiac MRI, anesthetized (1.5% (v/v) isoflurane) mice were placed into a magnetic resonance scanner (Bruker 9.4 T AVANCE III WB NMR-spectrometer, Bruker, Rheinstetten, Germany; Haberkorn et al., 2017), where an integrated heating pump kept body temperature of the mice constant. Vital parameters (heart rate, respiratory rate, and body temperature) were monitored by M1025 System (SA Instruments, Stony Brook, United States). Cine-FLASH-sequences were recorded during several cardiac cycles to measure left and right ventricular function and analyzed using ParaVision 5.1 (Bruker). Parametric maps of T1 and T2 values were recorded and calculated as published recently (Bonner et al., 2015; Haberkorn et al., 2017).

### Blood Analysis

Twenty-four hours after surgery mice were injected intraperitoneally with 10 mg/kg xylazine and 100 mg/kg ketamine. Blood was drawn from the retrobulbar plexus with a heparinized glass capillary and centrifuged for 10 min, 300 *g* and at 4°C. Plasma was obtained to measure Troponin T levels by a high-sensitivity immunoassay (Cobas, Basel, Switzerland). Furthermore, catecholamines were determined by 3-CAT-Research ELISA (LDN, Nordhorn, Germany) and serum amyloid P (SAP) component was tested with an ELISA Kit (GenWay Biotech, San Diego, United States). Plasma cytokines were measured using the BioPlexMouse Cytokines Th17-kit [IL-1ß, IL-6, IL-10, IL 17A, tumor necrosis factor alpha (TNFα) and interferon gamma (IFNγ; BioRad, Hercules, United States] and a BioPlex 200 reader (BioRad).

To analyze circulating leukocytes, the remaining cell pellet was transferred to flow buffer (Supplementary Table T2) at 4°C until further processing within 2 h. Erythrocytes were lysed by adding 4 volumes of ammonium chloride solution (Lysis Solution, Uniklinikum Düsseldorf, Germany) for 5 min at 4°C, washed using flow buffer, centrifuged (300 *g*, 10 min, 4°C) and the supernatant discarded. This process was repeated for three times. Cells were stained for 30 min in FcR-blocking solution (1:25 in flow buffer; Miltenyi, Bergisch Gladbach, Germany) containing fluorochrome-conjugated antibodies directed against CD45, F4/80, CD11b, Ly6C, Ly6G, CD 206 (BD Bioscience, Heidelberg, Germany; 1:100 in flow buffer); CD19, CD3 (Miltenyi; 1:100 in flow buffer). For intracellular staining of TNFα (BD Bioscience; 1:100 in FACS buffer), cells were treated using Cytofix/Cytoperm™ (BD). Cell viability was analyzed using 7-amino-actinomycin D (7-AAD; BioLegend).

All samples were measured using a Canto II flow cytometer (BD Bioscience). The related gating strategy is shown in the Supplementary Figure S4.

### Isolation of Cardiac Leukocytes

Preparation of cardiac leukocytes was performed as described previously (Bonner et al., 2012, 2013). Briefly, hearts were harvested and immediately transferred into PBS at 4°C for further preparations. To dissect the aortic arch surrounding tissue was removed. A cannula was inserted into the dissected aorta and hearts were perfused retrogradually with washing buffer (Supplementary Table T3) for 5 min at 4°C. Hearts were digested using a collagenase solution (Supplementary Table T4) by retrograde perfusion with a constant perfusion pressure of 80 mmHg using a Langendorff apparatus. To obtain cells for flow cytometry cardiac tissue was subsequently ground, cells were disintegrated and re-suspended in PBS containing 2% bovine serum albumin (BSA). Suspensions were centrifuged (300 *g*, 10 min, 4°C) and step-wise filtered through cell strainers with different mesh sizes starting with 100 μm, 70 μm and finally 40 μm (BD). After centrifugation (300 *g*, 10 min, 4°C) cells were stored in flow buffer at 4°C until further processing as described above within 2 h. The respective gating strategy is shown in the Supplementary Figure S5.

### Measurement of Splenic Leukocytes

To analyze splenic leukocytes, spleen was homogenized through a 100 μm cell strainer and transferred into flow buffer until further processing as described above.

### Measurement of Cardiac Cytokines

To measure cardiac cytokine levels, hearts were dissected, homogenized with tissue grinder, and lysed using the cell-lysis kit (Bioplex Cell Lysis Kit, BioRad) for 20 min at 4°C. After centrifugation (10,000 *g*, 4 min, 4°C), the pellet was discarded and protein concentration determined (DC BioRad assay). Cytokine concentrations were determined as described above using the BioPlex Mouse Cytokines Th17-kit.

### Quantitative Polymerase Chain Reaction

RNA was isolated from heart using the RNeasy Mini Kit (Qiagen, Hilden, Germany) and was reverse transcribed with the SuperScript IV VILO Master Mix with ezDNase synthesis kit (Thermo Fisher Scientific, Carlsbad, United States).

Quantitative polymerase chain reaction was performed using TaqMan Fast Advanced Master Mix and pre-manufactured primers and probes (all Thermo Fisher Scientific) for genes of interest, with glycerinaldehyde-3-phosphate-Dehydrogenase (GAPDH) as reference housekeeping gene on a ViiA7 real-time PCR system (Thermo Fisher Scientific).

Data were analyzed on the basis of the relative expression method with the formula: 2–ΔΔCT, where ΔΔCT = ΔCT (sample) – ΔCT (calibrator = average CT values of all samples), and ΔCT is the CT of the GADPH-housekeeping gene subtracted from the CT of the target gene (Klingenberg et al., 2007).

### Quantitative Western Blot Analysis

Hearts were dissected and frozen on liquid nitrogen and stored at −80°C until further use. Frozen hearts were homogenized for 20 sec and further on lysed in NP-40 lysis buffer [150 mM sodium chloride, 1% NP-40, 0.1% SDS, 1 mM EDTA, 50 mM Tris–HCl, pH 7.6, with protease inhibitor (Sigma Aldrich)] for 1 min with the Mixer Mill MM 400 (Retsch GmbH, Haan, Germany). Lysates were cleared by centrifugation at 11,000 *g* for 20 min at 4°C. Protein concentration was measured by the BCA Protein Assay Kit (Sigma-Aldrich). Western blotting was performed using 100 μg total protein onto a precast NuPAGE 4–12% Bis-Tris gel (Thermo Fisher Scientific) at 20 mA for 60 min followed by transfer (100 V for 40 min) to an Amersham Protran 0.2 μm nitrocellulose membrane (VWR International, Radnor, United States) with blotting buffer (125 mM Tris, 960 mM glycine). The total protein was stained with the Revert 700 Total Protein Stain Kit (LI-COR Biosciences, Lincoln, NE, United States). The membrane was blocked with Intercept (TBS) Blocking Buffer (LI-COR Biosciences). The following antibodies were applied in TBS-Tween (0.1%): NF-κB 1:1,000), Phospho-NF-κB (1:1,000), Toll-like Receptor 4 (all Cell Signaling Technology, Frankfurt, Germany, 1:1,000), Anti-beta-1 Adrenergic Receptor antibody (Abcam, Cambridge, United Kingdom, 1:1,000), IRDye 800CW goat anti-rabbit IgG (1:20.000) and IRDye 680RD Goat anti-Mouse IgG (all LI-COR Biosciences, 1:20.000). The detection was performed on an Odyssey Fc Imaging System with Image Studio Software (version 5.2, LI-COR Biosciences).

### Histology

Freshly dissected hearts were perfused with PBS to remove residual blood and fixed in 4% paraformaldehyde in PBS (Affymetrix, Santa Clara, United States) at 4°C. After 24 h, hearts were transferred to 15% sucrose in PBS for 8 h at 4°C. Subsequently, hearts were transferred to 30% sucrose in PBS, followed by embedding in TissueTek® O.C.T.™ Compound (TissueTek, Sakura Finetek, Staufen, Germany) in methylbutane (Sigma-Aldrich) on dry ice. Embedded hearts were cut starting from the apex up to the valvular plane and a total of seven sections (5 μm thick) at a respective distance of 60 μm each were cut and kept on glass slides for further processing. To visualize apoptotic cells in myocardial tissue, a TdT-mediated dUTP-biotin nick end labeling (TUNEL)-staining kit (Insitu Cell Death Detection Kit-TMR Red, Roche, Basel, Switzerland) in combination with DAPI staining (Sigma-Aldrich) was used, according to the manufacturer’s protocol. Fluorescence images were obtained using a laser scanning microscope (LSM 710, Zeiss, Oberkochen, Germany) with a 40x objective magnification. The total number of apoptotic cells on all seven sections from one heart were counted manually.

To evaluate cardiac muscarinic receptor density, mouse anti-CHRM2 (Thermo Fisher Scientific) incubated on tissue sections overnight at 4°C. Secondary antibodies conjugated with fluorophores AF555 (Thermo Fisher Scientific) and DAPI (Sigma), were incubated in the dark for 1 h at room temperature. Stainings were imaged with a confocal laser scanning microscope (LSM 880, Zeiss). CHRM2 and DAPI areas in heart sections were quantified with FIJI (NIH, Bethesda, United States).

### Survival Analysis

In a subset of mice (*n* = 11), long-term mortality was assessed. Echocardiography and blood sampling were performed before and 24 h after tMCAO surgery as described above. To reduce animal distress during prolonged observation mice received 0.05 mg/kg buprenorphine in 0.3 ml of lactate ringer solution (Ringer-Laktat®, Braun, Melsungen, Germany) s.c. and administered softened food by gastric gavage every 7 h. Sham-operated mice were treated accordingly. Survival and condition of mice were checked every 4 h determined using pre-specified scores by an investigator blinded to the study protocol. Moribund animals were euthanized and death was recorded for a total duration of 168 h.

### Statistical Analysis

Data is presented as mean ± SD throughout. Statistical significance for comparison of two groups was determined using the student’s *t*-test and multiple comparisons were analyzed by one-way analysis of variance (ANOVA) followed by Tukey’s *post-hoc* test to identify group differences in variance analysis using the GraphPad Prism software. Before application of parametric statistical tests, data was tested for normal distribution using the Shapiro–Wilk test. If the number of data points was lower than seven, the Shapiro–Wilk test is not applicable and data were statistically analyzed using the non-parametric Mann–Whitney-U test. A value of *p* < 0.05 was considered as statistically significant. Accuracy was calculated by four-field-matrix.

### Data Availability Statement

The data underlying this article will be shared on reasonable request to the corresponding author.

## Results

### tMCAO Induces Elevation of hsTnT

**Figure 1:**
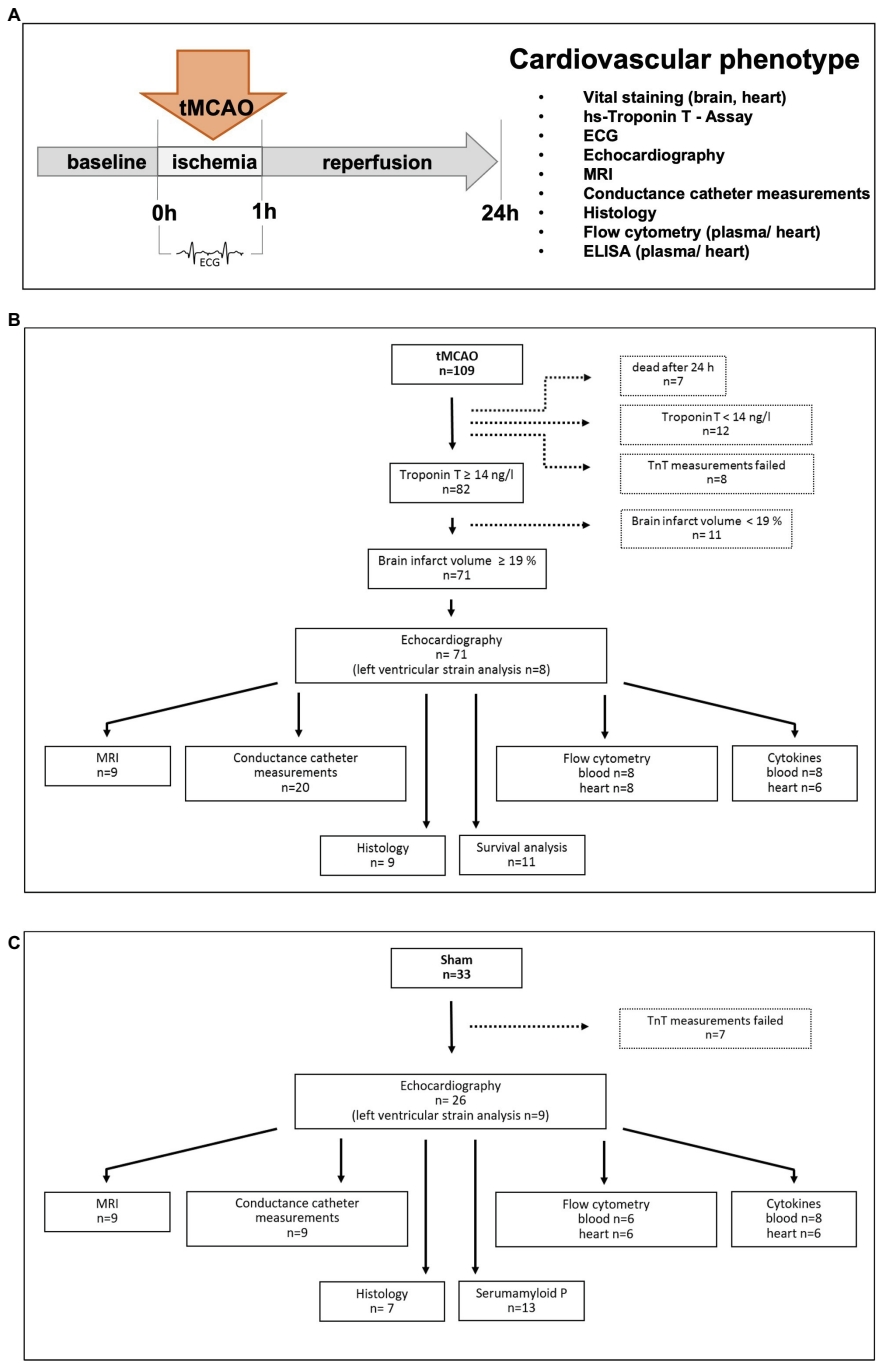
Experimental setup. **(A)** Schematic illustration of the experimental protocol: Male mice (12–14 weeks old) received right-sided transient (60 min) middle cerebral artery occlusion (tMCAO) and all methods indicated were applied after 24 h. **(B,C)** Flow charts of all (tMCAO and sham) experiments conducted, including drop outs and respective analysis.

Overall, 109 mice underwent tMCAO (60 min) procedure and subsequent the cardiovascular phenotype was characterized as indicated in Figure 1.

**Figure 2:**
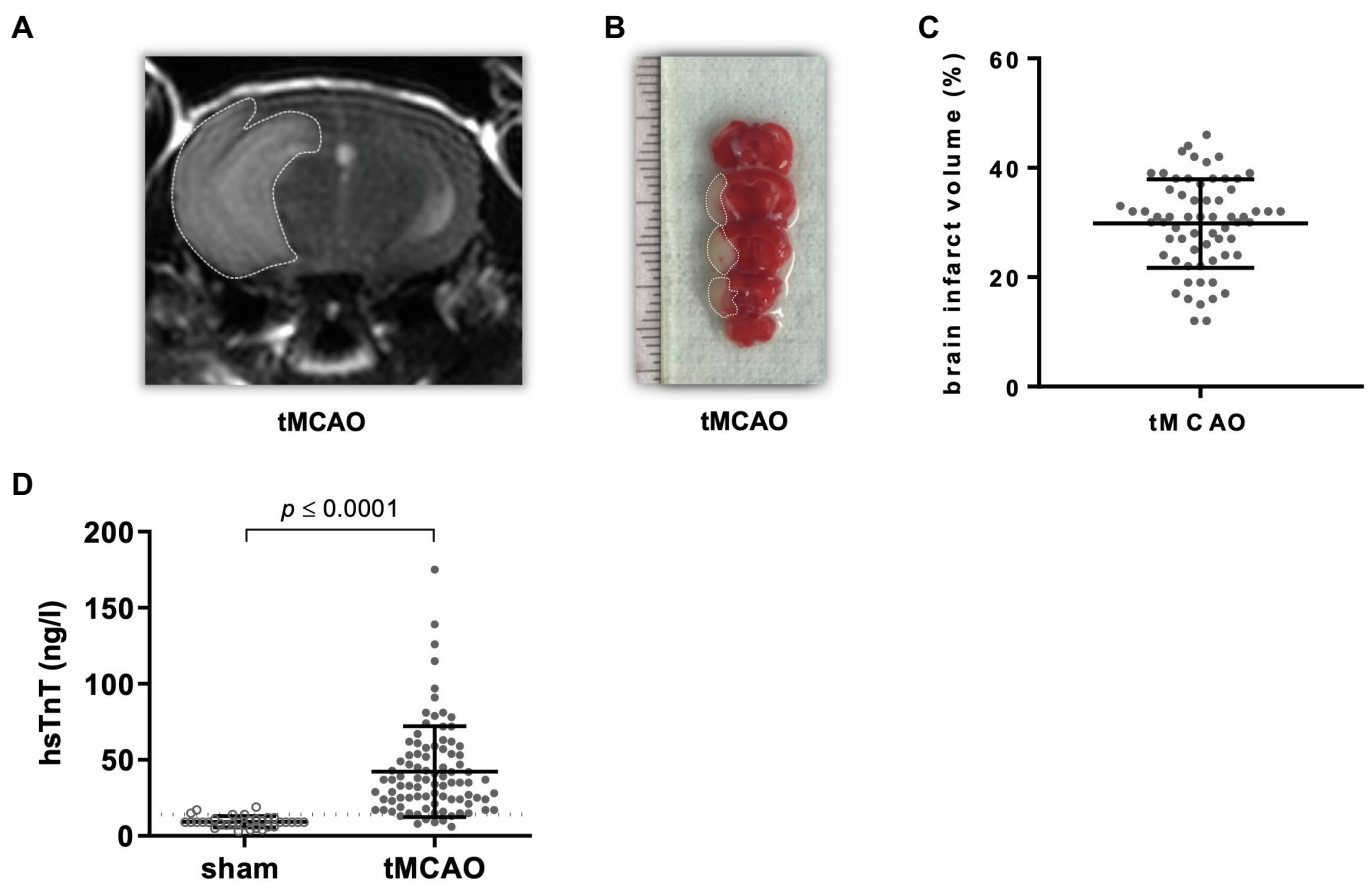
tMCAO causes a release of high-sensitive Troponin T (hsTnT). Male mice (12–14 weeks old) received right-sided tMCAO (60 min) or sham operation. Brain infarct size and circulating hsTnT were assessed 24 h thereafter. **(A,B)** Representative images of brain infarct volume determined by T2-weighted cerebral MRI and TTC-staining are shown. Dotted lines indicate ischemic regions. **(C)** Brain infarct volumes were determined in planimetric analysis of TTC staining (*n* = 71). **(D)** Circulating hsTnT levels in sham- and tMCAO-operated mice (*n* = 33 vs. 71; student’s *t*-test). Dotted line indicates the threshold level of 14 ng/l.

Stroke lesion volume was assessed 24 h after tMCAO using MRI and TTC-staining demonstrating 30 ± 8.1% lesion area relative to total brain volume (Figures 2A–C).

Neurologic-clinical examination of stroke-operated mice 1 hour after surgery deviated from the next time point 24 h after surgery: all mice were scored at least with two points, 65% of these animals showed a circulation motion (3 points). On the next day nearly all mice were scored with one point less than before. Circulating hsTnT values ranged between normal values <14–175 ng/L. In 75% of tMCAO, mice circulating hsTnT rose above the threshold of 14 ng/l (Figure 2D; Supplementary Figure S2). Brain lesion volume as calculated by TTC or by MRI did not correlate with plasma hsTnT levels (*r*^2^ = 0.01; Supplementary Figure S1A). Elevated hsTnT (>14 ng/L) was associated with an involvement of the insular cortex within the brain lesion volume with a diagnostic accuracy of 84.1% (sensitivity 93% and specificity 42%).

### tMCAO Leads to Cardiac and Hemodynamic Impairment 24 h After tMCAO

**Figure 3:**
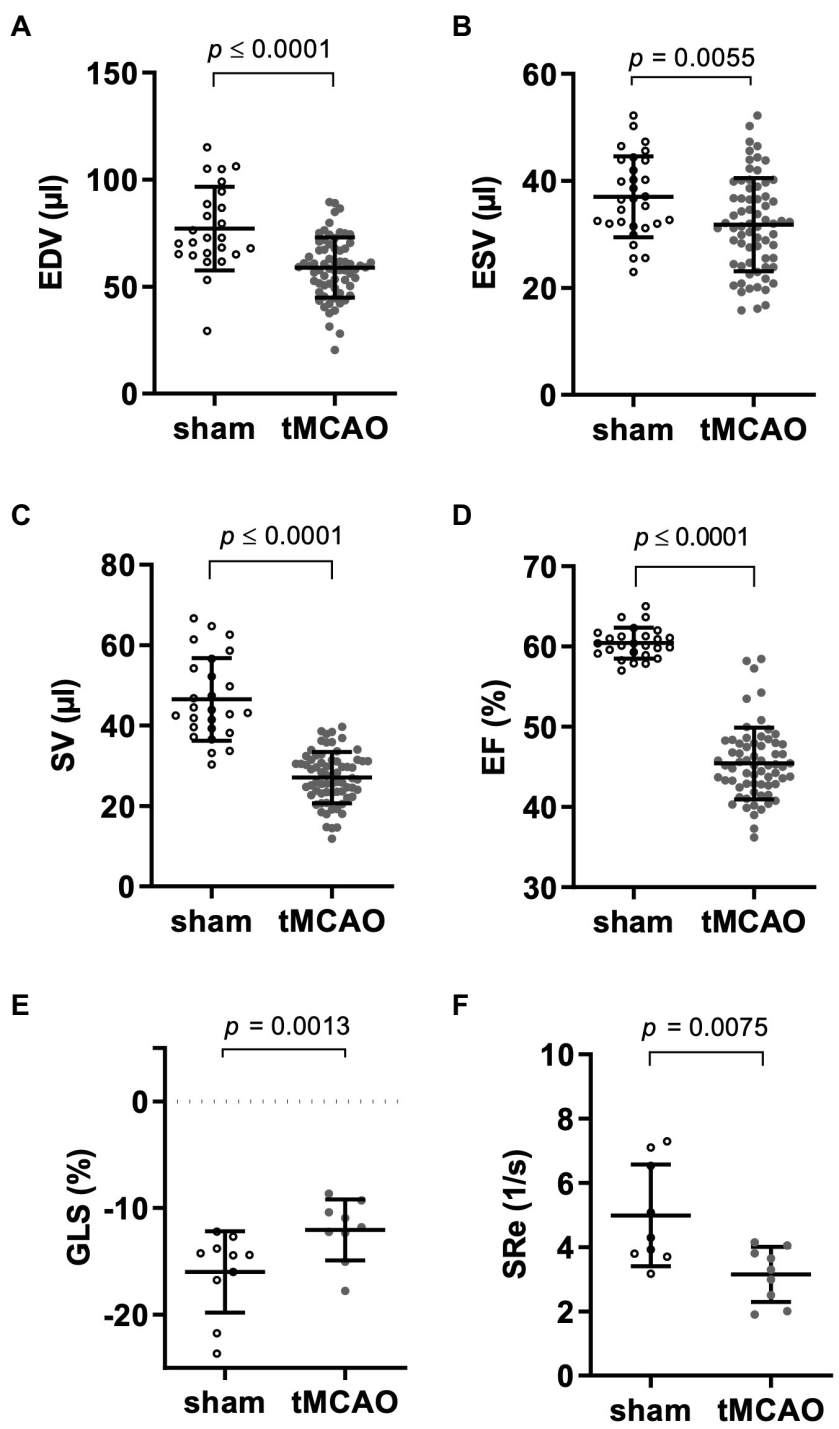
tMCAO causes predominant systolic left ventricular dysfunction. Mice received right-sided tMCAO (60 min) or sham operation and cardiac function was assessed by echocardiography 24 h thereafter. **(A)** End-diastolic (EDV; *n* = 25 sham vs. *n* = 71 tMCAO; student’s *t*-test), **(B)** end-systolic volumes (ESV; *n* = 30 sham vs. *n* = 71 tMCAO; student’s *t*-test), **(C)** stroke volume (SV; *n* = 26 sham vs. *n* = 71 tMCAO; student’s *t*-test), **(D)** ejection fraction (EF; *n* = 26 sham vs. *n* = 71 tMCAO; student’s *t*-test). Cardiac deformation analysis by speckle tracking determined **(E)** global longitudinal strain (GLS; *n* = 10 sham vs. *n* = 9 tMCAO; student’s *t*-test) and **(F)** early diastolic strain rate (SRe; *n* = 9 sham vs. *n* = 9 tMCAO; student’s *t*-test). *p*-values are given in the respective graphs.

In a next step, we investigated the alteration of cardiac function following tMCAO. Based on reduction of end-diastolic (EDV) and end-systolic volume (ESV), stroke volume (SV) and ejection fraction (EF) were reduced as measured by echocardiography (Figures 3A–D). In the context of proportionally reduced ESV and EDV and to exclude a relevant dehydration in our model, hematocrit and body weight were measured, yet no differences between sham and tMCAO were detected. Animals with tMCAO showed impaired global longitudinal strain (GLS) and reduced early diastolic strain rate (SRe) reflecting impaired systolic and diastolic cardiac deformation [Figures 3E,F and Supplementary Figure S12 (regional)] as assessed by echocardiography-based speckle tracking.

**Figure 4:**
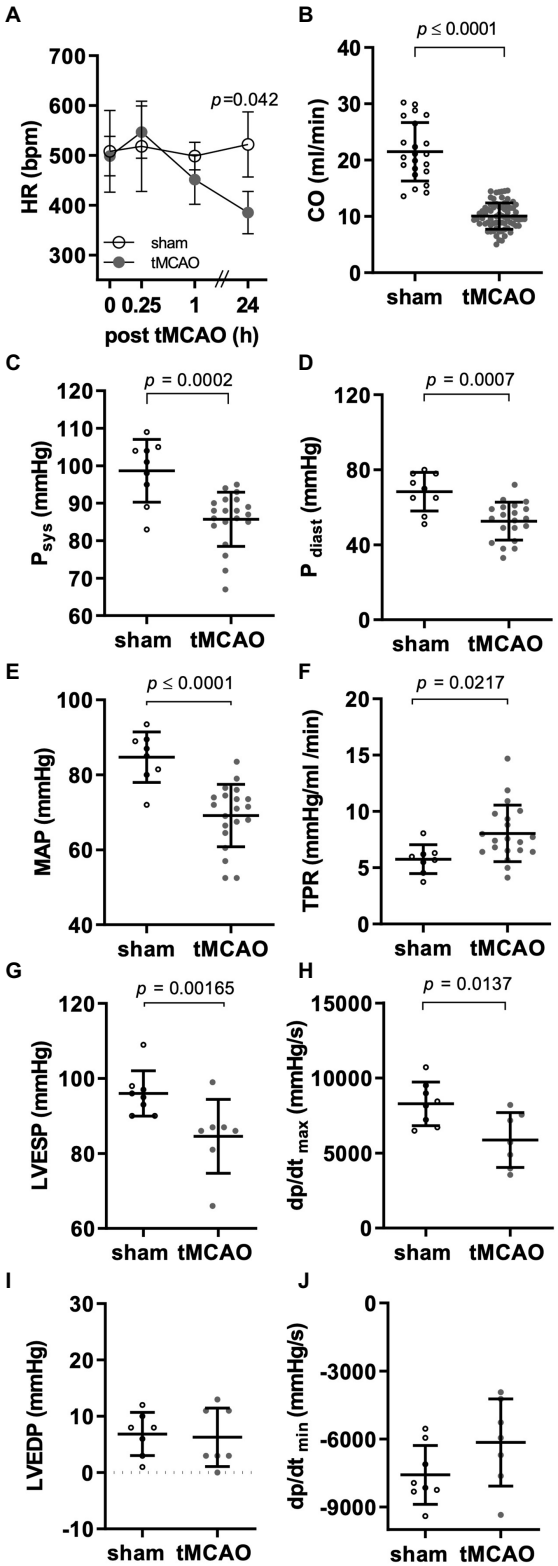
Cardiac and circulatory response to reperfused ischemic stroke. Male mice (12–14 weeks old) received right-sided tMCAO (60 min) or sham operation and were assessed by electrocardiogram, echocardiography, and conductance catheter measurements 24 h or indicated time points thereafter. **(A)** Heart rate at indicated time point following tMCAO as measured by electrocardiogram (*n* = 5 sham vs. *n* = 5 tMCAO, 2-way ANOVA). **(B)** Echocardiographic assessment of cardiac output (CO; *n* = 22 sham vs. *n* = 71 tMCAO; student’s *t*-test). Invasive conductance catheter measurements in the aorta determining **(C)** maximal (*P*_sys_; *n* = 9 sham vs. *n* = 20 tMCAO; student’s *t*-test), **(D)** minimal pressure (*P*_dias_; *n* = 9 sham vs. *n* = 20 tMCAO; student’s *t*-test), **(E)** mean arterial pressure (MAP; *n* = 8 sham vs. *n* = 20 tMCAO; student’s *t*-test), and **(F)** total peripheral resistance (TPR; *n* = 8 sham vs. *n* = 20 tMCAO, student’s *t*-test). Pressure–time analysis of **(G)** left ventricular end-systolic pressure (LVESP), **(H)** early dp/dt_max_ (*n* = 8 sham vs. *n* = 7 tMCAO; student’s *t*-test), **(I)** ventricular end-diastolic pressure (LVEDP; *n* = 7 sham vs. *n* = 7 tMCAO; student’s *t*-test), and **(J)** dp/dt_min_ (*n* = 8 sham vs. *n* = 7 tMCAO; student’s *t*-test).

tMCAO was accompanied by bradycardia compared to sham-operated animals (Figure 4A; Supplementary Figure S3). In conjunction with reduced cardiac stroke volume, this led to reduced CO (Figure 4B).

To corroborate these findings with hemodynamic measurements and calculation of peripheral resistance, we performed additional aortic and intraventricular pressure analyses. Aortic vessel maximal and minimal pressure were lower in tMCAO mice compared to sham-operated animals (Figures 4C,D). As a consequence, mean arterial pressure (MAP) was also reduced, while total peripheral resistance (TPR) increased following tMCAO (Figures 4E,F).

To analyze cardiac contraction and relaxation force, invasive pressure measurements were conducted. Left ventricular end-systolic pressure (LVESP) and left ventricular dp/dt_max_ values of tMCAO animals were lower compared to sham controls (Figures 4G,H). However, left ventricular end-diastolic pressure (LVEDP) and diastolic relaxation (dp/dt_min_) were impaired only by tendency (Figures 4I,J).

Heart rate variability analysis 24 h after surgery demonstrated significantly increased Mean NN and a tendential increased ration of Low/High Frequency bands in tMCAO mice in comparison to sham-operated mice (Supplementary Figure S14).

### tMCAO Leads to Expansion of Extracellular Space, Fluid Loss, and Diffuse Distribution of Apoptotic Cell Death in Myocardial Tissue Connected to Systemic and Local Inflammation

**Figure 5:**
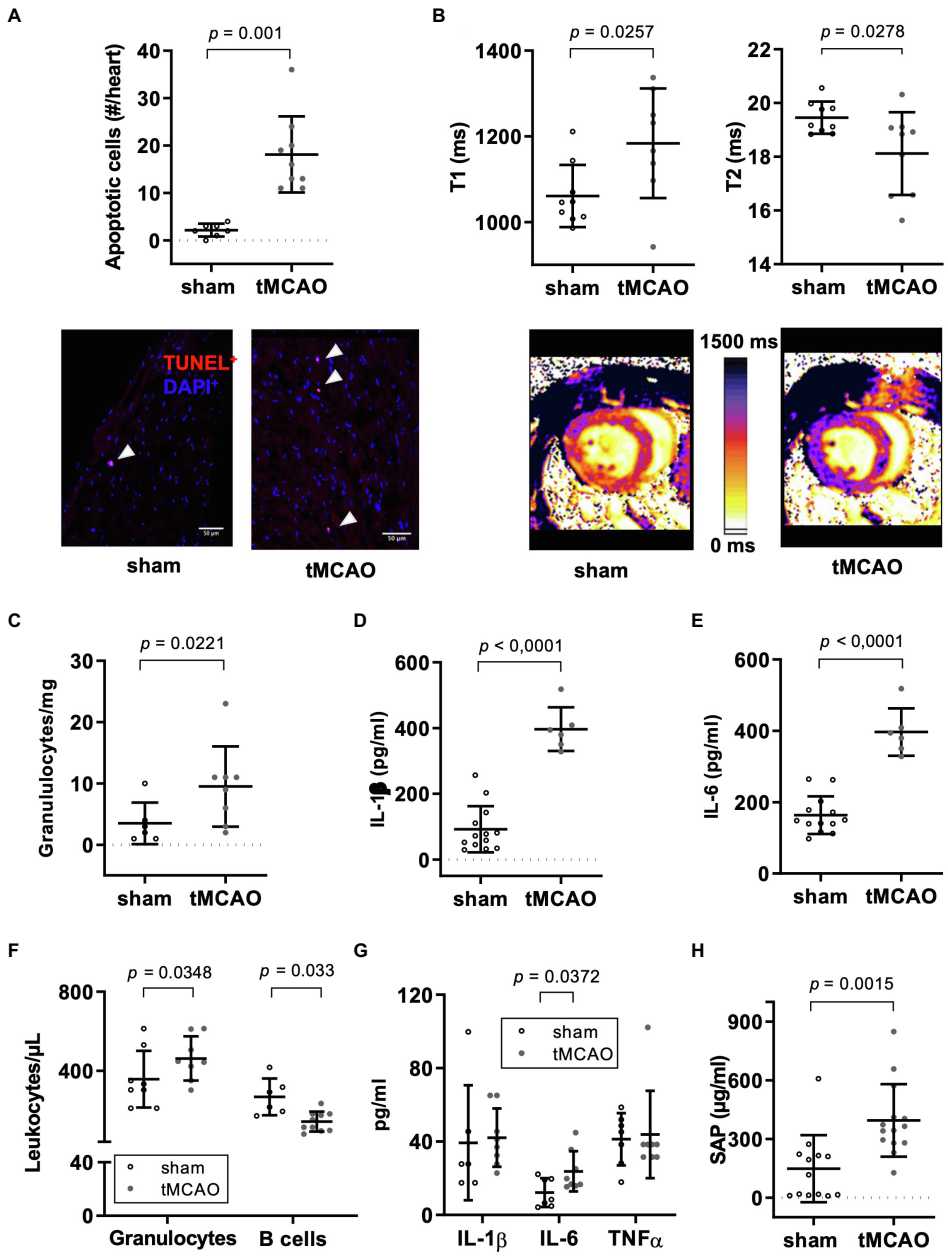
tMCAO leads to a systemic and local inflammatory response. Male mice (12–14 weeks old) received right-sided tMCAO (60 min) or sham operation hearts were analyzed by histology and MRI 24 h thereafter. Hearts and blood were additionally assessed by flow cytometry and multiplexed immune assay. **(A)** Determination of apoptotic cells by TUNEL-staining in global myocardial analysis. Apoptotic cells were counted in 7 sections with a distance of 60 μm. Quantitative analysis (above; *n* = 7 sham vs. *n* = 9 tMCAO; student’s *t*-test) and representative images of one field of view (below). Arrowheads indicate TUNEL^+^ cells (red). Scale bar is 50 μm. **(B)** Cardiac magnet resonance imaging with parametric mapping before and 24 h after right-sided tMCAO (60 min; T1: *n* = 9 sham vs. *n* = 8 tMCAO, T2: *n* = 9 sham vs. *n* = 9 tMCAO; student’s *t*-test). Global myocardial quantitative analysis (above) and representative images (below) are shown. **(C)** Flow cytometric analysis of granulocyte numbers in the heart (cells/mg heart tissue; *n* = 6 sham vs. *n* = 8 tMCAO; student’s *t*-test). Determination of **(D)** IL-1β and **(E)** IL-6 in lysates from hearts (*n* = 13 sham vs. *n* = 6 tMCAO; student’s *t*-test). **(F)** Flow cytometric analysis of circulating leukocyte subpopulations (granulocytes: *n* = 8 sham vs. *n* = 8 tMCAO, B cells: *n* = 6 sham vs. *n* = 10 tMCAO; student’s *t*-test). **(G)** Plasma cytokine (IL-1β: n = 6 sham vs. *n* = 8 tMCAO, IL-6 and TNFα: *n* = 7 sham vs. *n* = 8 tMCAO; student’s *t*-test) and **(H)** SAP concentrations as determined by immunoassays (*n* = 13 sham vs. *n* = 14 tMCAO; student’s *t*-test).

Considering the elevated circulating hsTnT levels, we further characterized the effect of tMCAO on myocardial tissue by histology. Indeed, tMCAO mice exhibited increased abundance of diffusely distributed apoptotic (TUNEL^+^) cells relative to controls (Figure 5A). We found no histological evidence of significant necrotic areas in TTC staining.

To further characterize myocardial tissue under in- vivo conditions, we performed MRI with relaxometry (T1- and T2-mapping) 24 h after tMCAO for analysis of myocardial water content and connective tissue (Haberkorn et al., 2017; Veltkamp et al., 2019). T1 relaxation time increased in a non-regional but global manner, most likely indicating global expansion of extracellular space, while T2 relaxation time decreased suggesting that there is a fluid loss in cardiac tissue in tMCAO compared to controls (Figure 5B). MRI-based evaluation of right ventricular function and proton density in pulmonary tissue revealed neither differences in volumes or function nor in incidence of pneumonia in tMCAO mice compared to controls (Supplementary Figure S3C).

To address the impact of systemic inflammation on the observed structural changes, we analyzed inflammatory markers in cardiac and splenic tissue. Numbers of resident granulocytes within myocardial tissue increased 24 h after tMCAO (Figure 5C); however, frequency of monocytes and lymphocytes did not change (Supplementary Figure S7). In addition, concentration of the pro-inflammatory cytokines IL-1β and IL-6 nearly doubled in heart tissue of tMCAO compared to sham-operated controls (Figures 5D,E), while IL-10 did not differ between these groups (Supplementary Figure S9A).

Quantitative analysis of TLR-4 gene expression demonstrated a significant upregulation (Supplementary Figure S9B), while key adhesion molecules (VCAM-, ICAM-1, and P-selectin) showed a trend toward higher expression (Supplementary Figures S9C–E). However, on protein level, TLR-4 receptor was not detectable. Downstream signaling molecules NF-ĸB and phosphorylated NF-ĸB did not differ in heart tissue between sham and tMCAO mice. tMCAO animals demonstrated increased circulating granulocyte counts and decreased B lymphocyte numbers compared to sham-operated controls (Figure 5F). Regarding neutrophil sub-populations in circulation, we observed a decreased expression level of CD206 in tMCAO mice indicating a higher N1/N2 ratio following stroke (Supplementary Figure S6C). However, number of monocytes, distribution of monocytes sub-populations, and T lymphocyte counts did not change (Supplementary Figures S6A,B,D). Circulating levels of the pro-inflammatory cytokine IL-6 and SAP were increased while IL-1β and TNFα levels did not differ between tMCAO mice and sham-operated controls (Figures 5G,H). Also, IL-10 did not change (Supplementary Figure S8). Levels of IL-17A and IFNγ were below the limit of quantification.

Also, in splenic tissue granulocytes were increased, while T cells were decreased 24 h after tMCAO (Supplementary Figure S10).

Of note, plasma concentrations of epinephrine, norepinephrine, and dopamine were increased only by tendency in tMCAO animals suggesting a minor role, if at all, for catecholamines (Supplementary Figure S11). Beta-1 Adrenergic protein expression and muscarinic receptor density in heart tissue did not differ in tMCAO compared to sham (Supplementary Figure S13).

### Troponin Elevation and Reduced Cardiac Output 24 h After tMCAO Indicate Increased Mortality at Long-Term Follow Up

**Figure 6:**
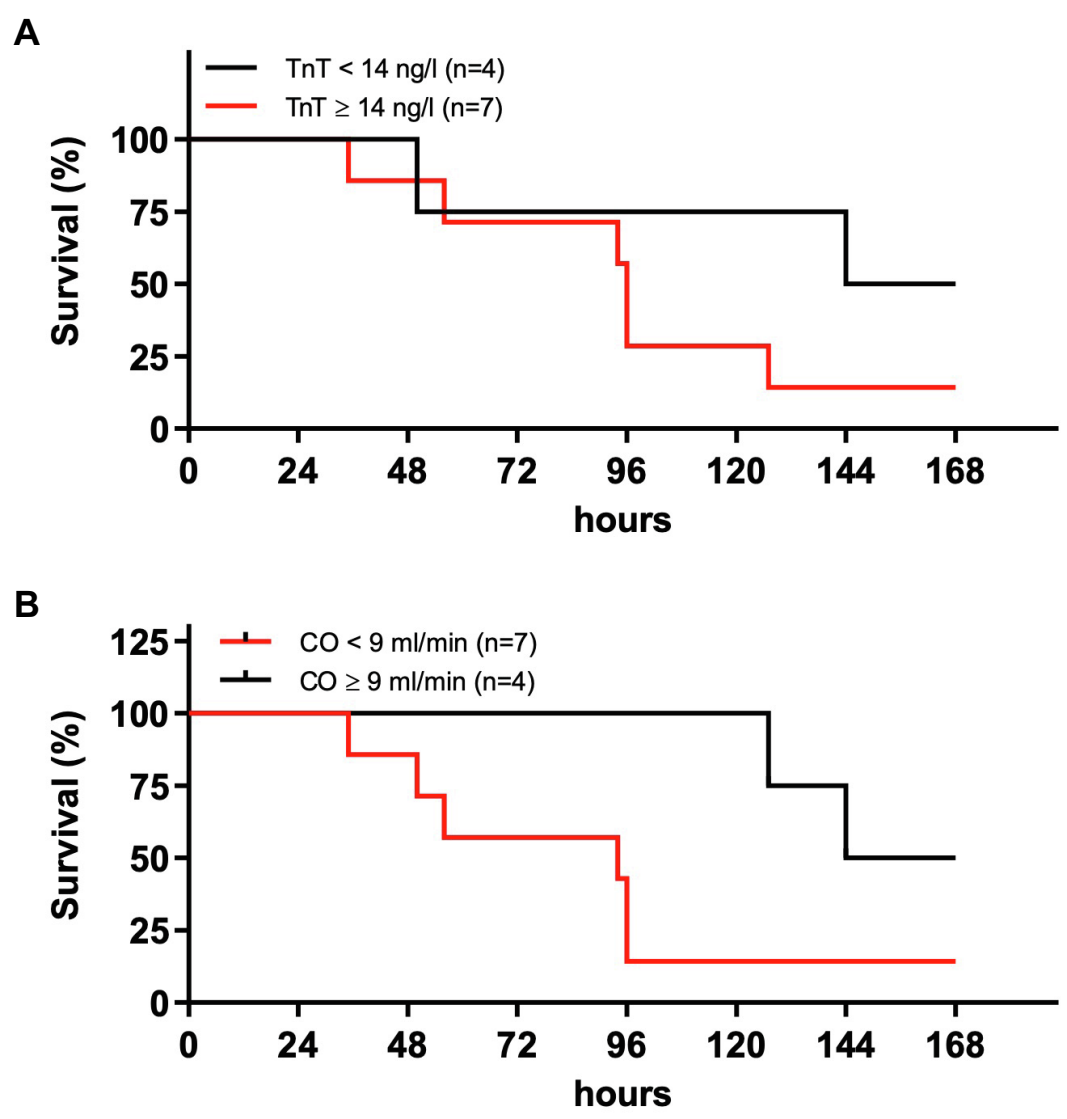
Early high-sensitive troponin levels and cardiac output predict mortality. Eleven male mice (12–14 weeks old) received right-sided tMCAO (60 min) and hsTnT and cardiac output (CO) was assessed 24 h later by immune assay or echocardiography, respectively. Survival was monitored for a total duration of 168 h. **(A)** Survival curve of mice dependent of hsTnT concentration below (black) and above (red) 14 ng/L. **(B)** Survival curve of mice dependent of CO below (red) and above (black) 9 ml/min.

Survival analysis revealed that elevated hsTnT levels at 24 h after tMCAO predicted premature death within the observation period of 7 days (Figure 6A). Functional impairment in terms of heart failure as expressed by reduced CO below 9 ml/min measured 24 h after surgery predicted increased mortality (Figure 6B).

## Discussion

Our study shows that right-sided tMCAO in mice leads to a profound cardiac injury with circulatory alterations leading to acute heart failure. In detail, we found (1) a 4-fold increase in circulating hsTnT which predicted mortality, (2) the release of hsTnT did not derive from myocardial infarction but rather from scattered myocardial injury in conjunction with local inflammation, apoptotic cell death, reduction of fluid volume and expansion of extracellular space in cardiac tissue, (3) tMCAO caused bradycardia as well as acute heart failure with systolic and diastolic left ventricular dysfunction, resulting in severe impairment of cardiac output, and (4) tMCAO led to an inflammatory response syndrome with systemic and cardiac inflammation.

These results mirror typical clinical findings of patients after AIS. HsTnT was elevated above the reference limit in a significant proportion of mice that were not affected by atherosclerosis. This is in line with a recent clinical study, reporting the absence of a coronary culprit lesion in the majority of the AIS patients with elevated hsTnT levels (MINOCA; Mochmann et al., 2016). Initial hsTnT levels did not correlate to the volume of cerebral stroke, a finding recently confirmed in a large clinical trial (Krause et al., 2017). This trial showed a clear dependency of hsTnT elevation and involvement of the right anterior insular cortex (van der Bilt et al., 2009). In our model hsTnT elevation (≥14 ng/L) likewise was associated with infarction of the right insular cortex. Similar to our experimental study hsTnT correlated with reduced survival after AIS in patients (Anders et al., 2013).

The stroke insult size, on average of 30%, demonstrated relatively high variability (8.1%). This is reflected in the clinical symptoms of these mice and also described by Clark et al. Clark et al. (1997). The neurological improvements 1 day after surgery indicate that mice have rapid compensation mechanisms leading to a very prompt recovery phase.

In contrast to previous findings, in murine models with predominant tachycardia at later stages after tMCAO (Bieber et al., 2017), our experimental model produced a robust bradycardia in the acute phase following tMCAO. It is known that bradycardia, AV-blocks, and altered heart rate variability occur frequently after right-sided AIS with involvement of the insular region (Christensen et al., 2005; Abboud et al., 2006) that plays a paramount role in autonomic balance. In rodents, the cardiac afferent input is relayed to the posterior insula *via* the thalamus and integrated with the information received from higher cortical centers in the rostroventral insula (Oppenheimer and Cechetto, 1990). Thus, functional extinction of one insula results in dominance of the contralateral activity. Heart rate variability (HRV) analysis by ECG recordings during induction of tMCAO until 24 h of follow up revealed hints of autonomic disbalance. The observed changes point to the direction of a parasympathetic dominance. However, the meaning of HRV read-outs are controversy discussed in the literature. (De Raedt et al., 2015). Mice are predominantly believed to be of the sympathetic type (due to low tolerance to external stimuli; e.g., handling), which was most likely counterbalanced by our model (Gehrmann et al., 2000).

This leads to parasympathetic effects in the early phase after AIS and might explain our observed phenotype that was not related to changes in muscarinic and beta adrenergic receptor density (Oppenheimer and Cechetto, 2016). However, bradycardia has also been described in models of permanent left-sided MCAO (Lubjuhn et al., 2009). Thus, species, model (reperfused vs. non-reperfused) and index-time after tMCAO might affect extent of bradycardia.

The present study shows a consistent functional cardiac phenotype with acute heart failure due to systolic and diastolic dysfunction with bradycardia after right sided tMCAO. Acute heart failure has been described after myocardial infarcton with non-obstructive coronary arteries (MINOCA), systemic inflammatory response syndrome (SIRS) and Takotsubo cardiomyopathy. The pathomechanism of acute heart failure after reperfused stroke is not known. However, Takotsubo cardiomyopathy was identified to mirror some features of acute heart failure after AIS. Physical or emotional stress is considered to induce a significant catecholamine surge leading to an acute coronary syndrome-like clinical presentation including ECG changes and wall motion abnormalities without obstructive coronary artery disease (Templin et al., 2015; Yoshikawa, 2015). In our model of tMCAO, we did not find elevated circulating catecholamines in the acute phase, contrasting the finding of increased plasma catecholamines in long-term observation after stroke (Clark et al., 1997). Supporting the minor role of catecholamines in our model, we observed a profound bradycardia instead of a tachycardia. This finding is of great interest since heart failure as observed in our model would normally lead to a compensatory tachycardia to maintain CO. Yet, both reduced stroke volume and bradycardia synergistically caused reduced CO.

As a potential circulating mediator of AIS-induced cardiac dysfunction, we found a systemic inflammatory response with increased levels of granulocytes, SAP and IL-6. Of note, neutrophil polarisation towards N1 and reduction of N2 has emerged as crucial for cardiac injury post-myocardial infarction (Ma et al., 2016) and is also described in brain inflammatory response after stroke (Cuartero et al., 2013). We observed a decrease of CD206 expression on circulating neutrophils suggesting an elevated in N1/N2-ratio and indicating a more inflammatory phenotype of neutrophils in tMCAO animals. All these findings also mirror clinical observations of a profound systemic inflammation with elevated CRP and IL-6 predicting long-term mortality after AIS (Offner et al., 2006; Pusch et al., 2015). Cellular inflammation was accompanied by SIRS with reduced mean arterial pressure, LVESP as well as reduced contraction and relaxation parameters dp/dt_max_ and dp/dt_min_. These results also resemble hemodynamic features of sepsis models in mice, as we have shown previously (van de Sandt et al., 2013). It is also known, that a stroke-induced immunodepression, mediated by the autonomous nervous system, leads to secondary complications such as pulmonary diseases (Dirnagl et al., 2007). Yet, we could not observe changes in right ventricular function or proton density in lung tissue of tMCAO mice compared to sham controls. In our model, the observed hemodynamics most likely reflect an acute systolic myocardial failure with systemic vascular failure of blood pressure maintenance. Regarding the immunosuppression, the activity of the peripheral nervous system might be mediate such a rapid response *via* immune reflex.

Also, Toll-like receptor (TLR)-4 has been ascribed an aggravating function in inflammation after stroke and myocardial infarction resulting in the development of heart failure (Yang et al., 2016). Indeed, in our study TLR-4 was markedly upregulated indicating early activation of innate immune responses while expression of adhesion molecules did not significantly differ.

Interestingly, long term cardiac functional outcome was impaired in mice that carried an endothelial-specific deficiency for mircoRNA-126. However, this study did not focus on short-term stroke-associated effects on cardiac dysfunction (Chen et al., 2017a).

However, in our study, we observed a decreased cardiac output not only due to a reduced stroke volume but also due to bradycardia as well as maintained peripheral resistance with constant hematocrit and body weight. Yet, the influence of autonomic vascular tone, venous return and fluid resuscitation cannot be ruled out. These latter observations are unique features of our model and seem not to resemble features of SIRS, Takotsubo cardiomyopathy or myocardial infarction.

We here report specific tMCAO-induced alterations of myocardial tissue: increased levels of apoptosis, a global expansion of extracellular space, fluid loss and inflammation within myocardial tissue after tMCAO. In combination with elevation of hsTnT, these findings indicate a specific pattern of myocardial injury, however, not resembling myocardial infarction type 1 or 2. Myocardial and systemic inflammations were accompanied by a unique adaptation of systemic circulation with maintained total peripheral resistance despite of an AIS-induced systemic inflammatory response. The exact mechanisms of how these systemic factors impact on myocardial function require further clarification.

Such studies should identify novel targets to modulate immune and neurogenic reflexes and responses following AIS. Here, we provide a valuable experimental model to further study the complex systemic and local interaction of the brain and the heart in reperfused AIS, that is applicable to transgenic mice to study relevant signaling cascades of heart, vessels, autonomic nerves and brain.

To conclude, reperfused ischemic stroke leads to specific cardio-circulatory alterations that are characterized by acute heart failure with reduced stroke volume, bradycardia and accompanied by systemic and local inflammatory responses.

## Data Availability Statement

The raw data supporting the conclusions of this article will be made available by the authors, without undue reservation.

## Ethics Statement

The animal study was reviewed and approved by the State Agency for Nature, Environment and Consumer Protection (LANUV), Leibnizstr. 10 Hauptsitz, 45659 Recklinghausen (registration number: AZ 84-02-04-14.A338).

## Author Contributions

LV, FN, MG, NG, SJ, and FB: conception and study design. LV, FN, CA, CH, AL, HE, LC, and UF: conduction of experiments. LV, FN, MG, LC, UF, MK, NG, SJ, and FB: interpretation of data. LV, FN, MG, GW, MK, NG, SJ, FB, and TR: draft of manuscript. LV, MG, FN, CA, CH, AL, HE, GW, LC, TR, UF, MK, NG, SJ, and FB: final approval. All authors contributed to the article and approved the submitted version.

## Funding

This work was supported by a Grant from the Forschungskommission of the Medical Faculty of the Heinrich-Heine-University Duesseldorf [9772584 to SJ and FB] and Deutsche Forschungsgemeinschaft (DFG; GRL BO-4264/1-1 to FB; CRC Grant No. 236177352 - SFB1116 projects B06 to MK, B09 to NG, B10 to UF, GEROK stipend to FN, CRC Grant No. 397484323 - TRR259 project B03 to FB and UF).

## Conflict of Interest

The authors declare that the research was conducted in the absence of any commercial or financial relationships that could be construed as a potential conflict of interest.

## Publisher’s Note

All claims expressed in this article are solely those of the authors and do not necessarily represent those of their affiliated organizations, or those of the publisher, the editors and the reviewers. Any product that may be evaluated in this article, or claim that may be made by its manufacturer, is not guaranteed or endorsed by the publisher.
