# Supplementary material for: Acute Heart Failure After Reperfused Ischemic Stroke: Association With Systemic and Cardiac Inflammatory Responses
Source: Front Physiol. 2021 Dec 21;12:782760. doi: 10.3389/fphys.2021.782760 (PMC8724038; doi:10.3389/fphys.2021.782760)
Supplement: Supplementary file 1 [file Data_Sheet_1.docx]

**SUPPLEMENTAL MATERIAL** to:

**Acute Heart Failure after Reperfused Ischemic Stroke: Association with Systemic and Cardiac Inflammatory Responses**

Stroke-induced Acute Heart Failure

Lilian Vornholz^a,b^, DVM, Fabian Nienhaus^a^, MD, Michael Gliem^b^, MD, Christina Alter^c^, PhD, Carina Henning^d^, PhD, Alexander Lang^a^, PhD, Hakima Ezzahoini^a^, Georg Wolff^a^, MD, Lukas Clasen^a^, MD, Tienush Rassaf^e^, MD, Ulrich Flögel^a,c,f^, PhD, Malte Kelm^a,f^, MD, Norbert Gerdes^a^, PhD, Sebastian Jander^b*^, MD, Florian Bönner^a*^, MD

^a^ Division of Cardiology, Pulmonology, and Vascular Medicine, Medical Faculty, University Hospital Düsseldorf, Düsseldorf, Germany

^b^ Department of Neurology, Medical Faculty, University Hospital Düsseldorf, Düsseldorf, Germany

^c^ Experimental Cardiovascular Imaging, Department of Molecular Cardiology, Medical Faculty, Heinrich-Heine-University Düsseldorf

^d^ Institute of Metabolic Physiology, Department of Biology, Heinrich-Heine University, Düsseldorf, Germany

^e^ Department of Cardiology and Vascular Medicine, West German Heart and Vascular Center, Medical Faculty, University Hospital Essen, Essen, Germany.

^f^ Cardiovascular Research Institute Düsseldorf (CARID), Heinrich Heine University, Düsseldorf, Germany

* These authors have contributed equally to this work

Correspondence to: Florian Bönner

Division of Cardiology, Pulmonology, and Vascular Medicine

Medical Faculty, University Hospital Düsseldorf

Moorenstr. 5, 40225 Düsseldorf, Germany

Phone: +49 (0)211 81 18278

[florian.boenner@med.uni-duesseldorf.de](mailto:florian.boenner@med.uni-duesseldorf.de)

**1) Supplemental Figures**

**
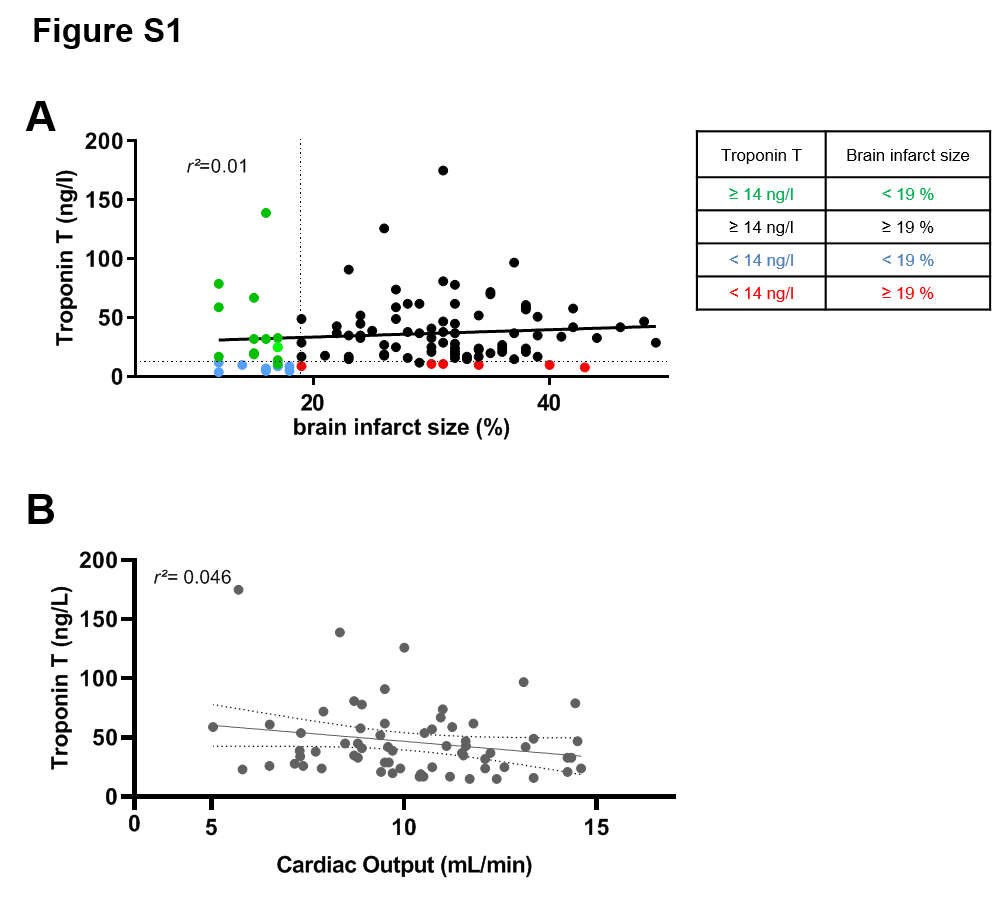
**

**Figure S1. Troponin T levels do not correlate neither to the volume of brain infarct nor to Cardiac Output 24 hours after ischemic stroke in mice**.

Male mice (12 - 14 weeks old) received right-sided tMCAO (60 min) or sham operation. High-sensitive Troponin T concentration in the plasma and brain infarct size and cardiac output were determined 24 h later. **A)** Troponin T concentration was plotted against brain infarct size. Colors indicate cut-off criteria for inclusion of mice for further investigations (black dots). **B)** Troponin T concentration was plotted against cardiac output.

 **Figure S2**

**Figure S2. tMCAO causes a release of high-sensitive Troponin T (hsTnT)**

Distribution of hsTnT levels in all tMCAO-treated mice (n=94) The red line indicates the threshold level of 14 ng/l.

**
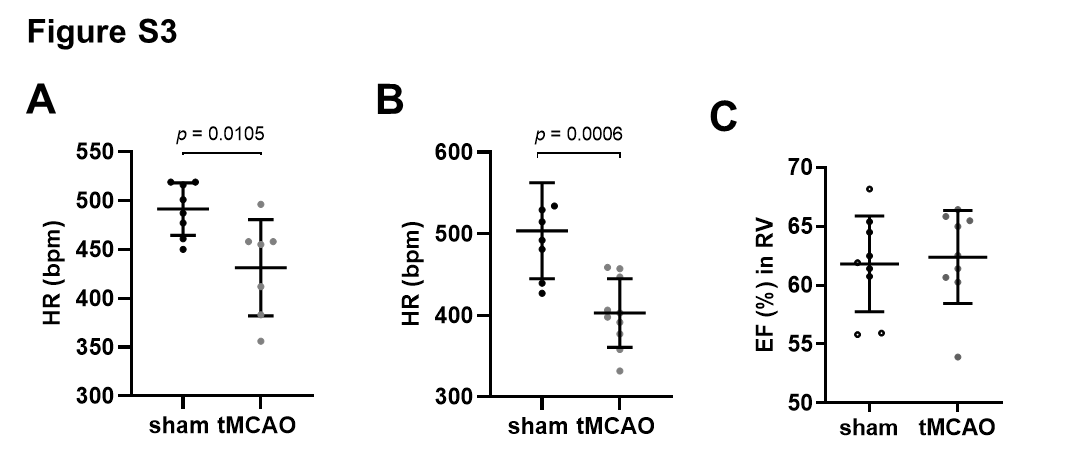
**

**Figure S3. tMCAO causes a robust bradycardia 24 h after surgery and no changes in right ventricular function**

Male mice (12-14 weeks old) received right-sided tMCAO (60 min) or sham operation. Heart rate was assessed 24 h later by electrocardiogram while **A)** conductance catheter measurements and **B)** echocardiography. **C)** MRI-based measurements of right ventricular function.

**
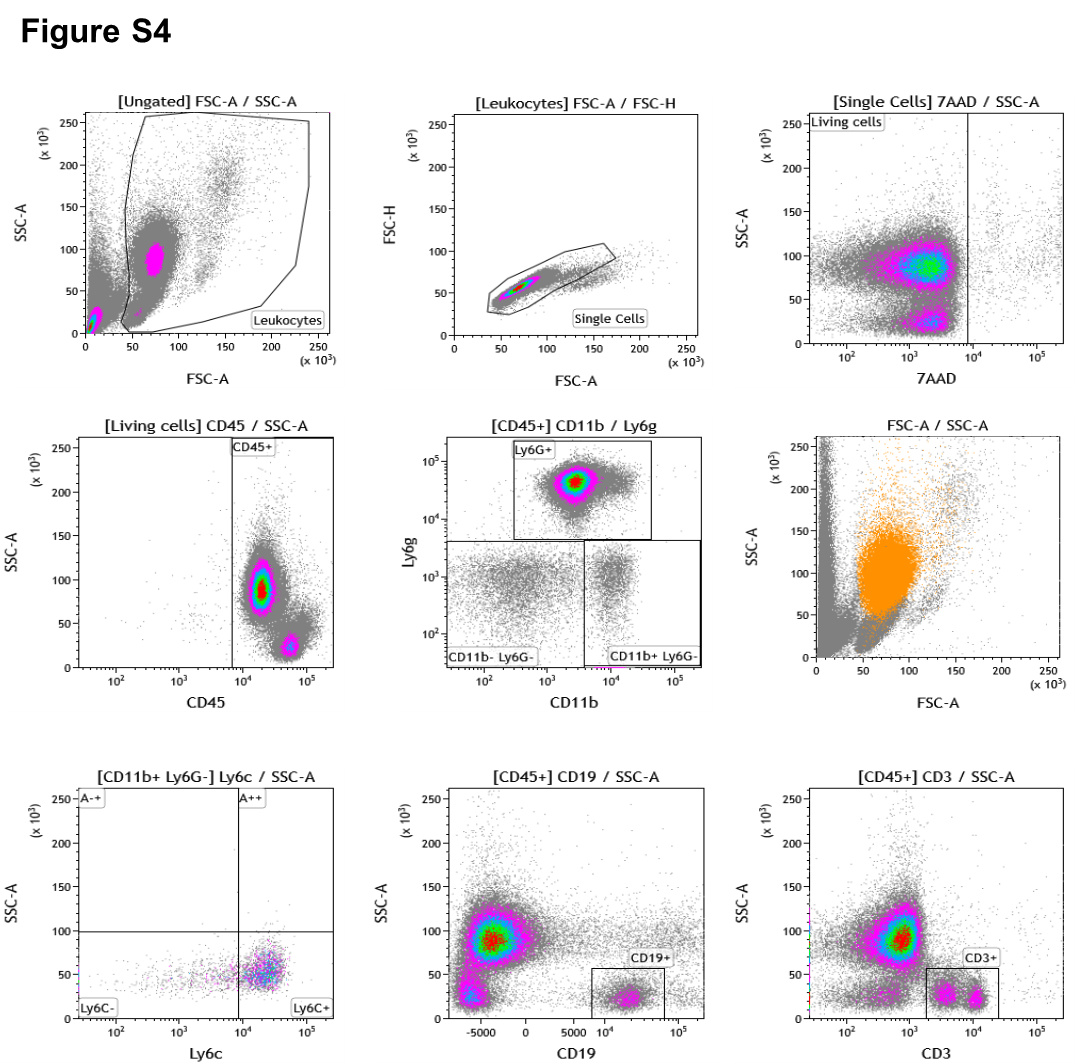
**

**Figure S4.** **Gating strategy of flow cytometry analysis for circulating leukocytes**

Blood cells were stained with Fluorochrome-conjugated antibodies against CD45, CD11b, Ly6G, Ly6C, CD19 and CD3.

**
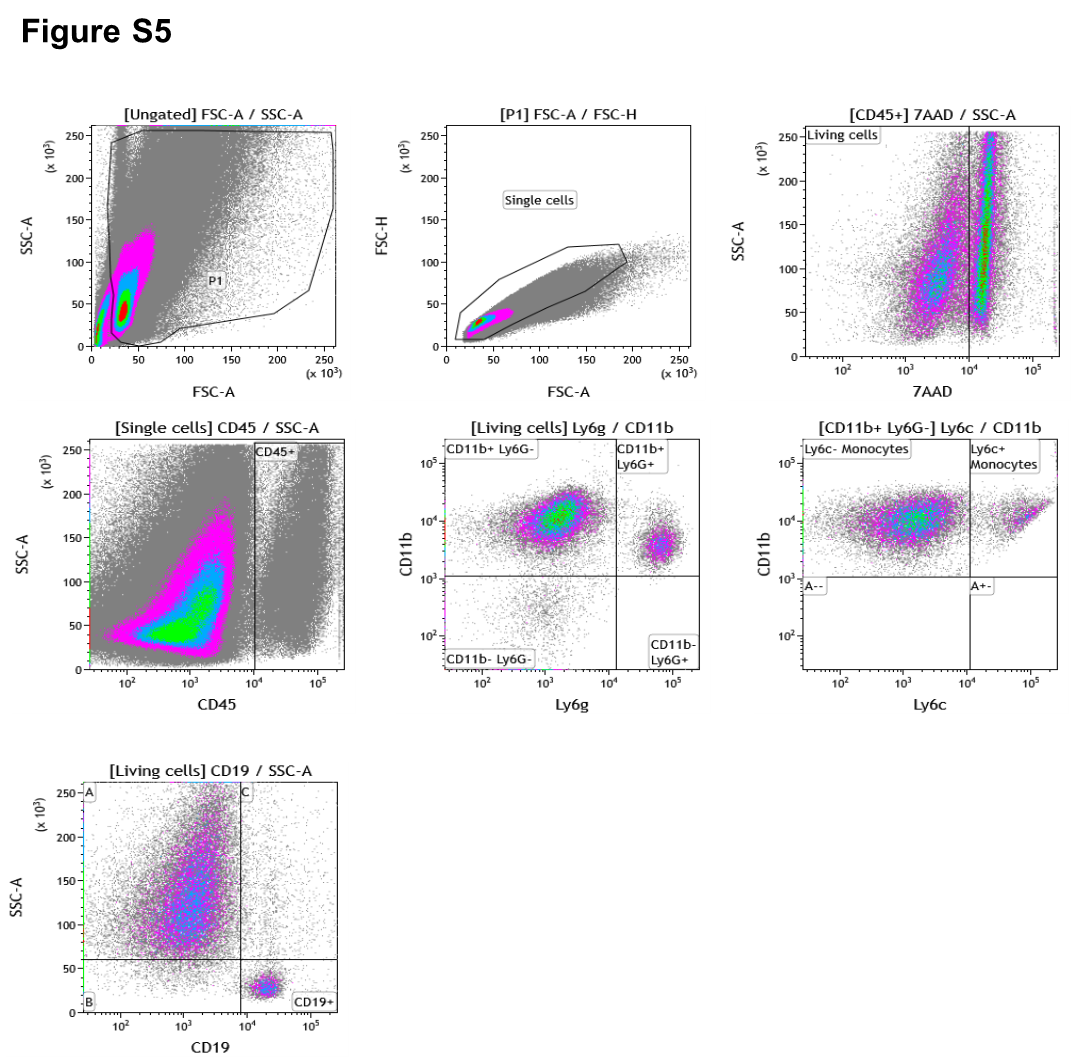
**

**Figure S5.** **Gating strategy of flow cytometry analysis for cardiac leukocytes**

Leukocytes of myocardial tissue were isolated by enzymatic digest and stained with Fluorochrome-conjugated antibodies against CD45, CD11b, Ly6G, Ly6c and CD19.


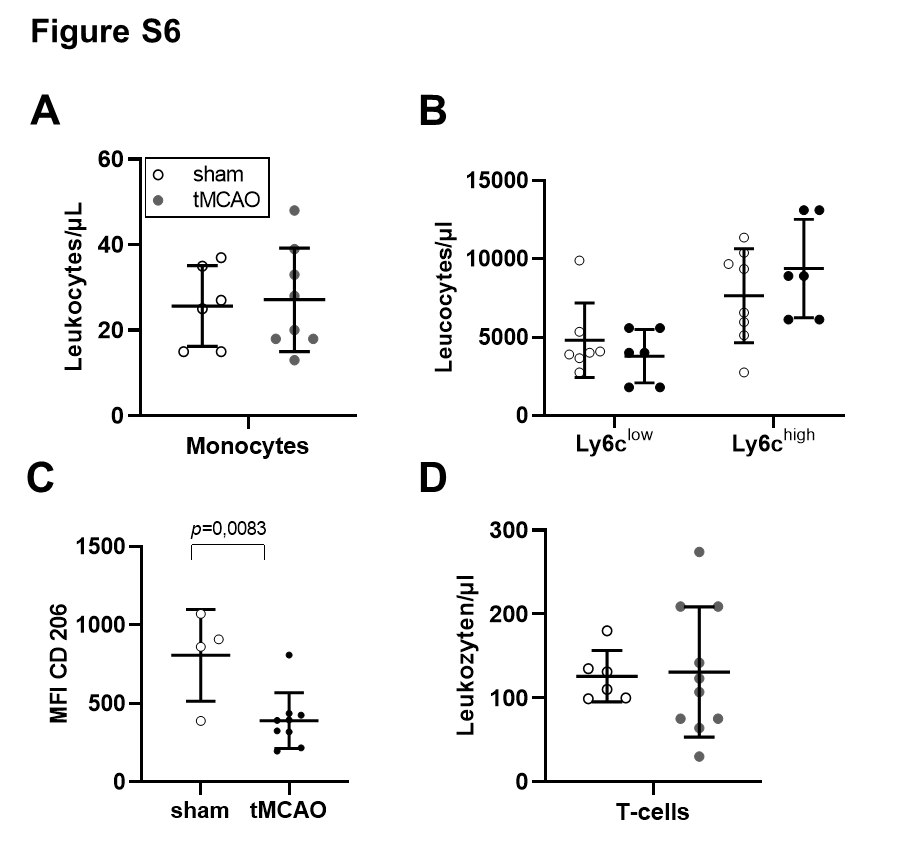


**Figure S6.** **Circulating monocytes and T cells do not differ after tMCAO**, **however neutrophils appeared N1 polarized**

Male mice (12-14 weeks old) received right-sided tMCAO (60 min) (filled circles) or sham (open circles). **A)** Monocytes, **B)** monocyte sub-populations, **C)** neutrophil polarization based on CD206 (marker for N2 neutrophils) expression and **D)** T cells were assessed by flow cytometry.

**
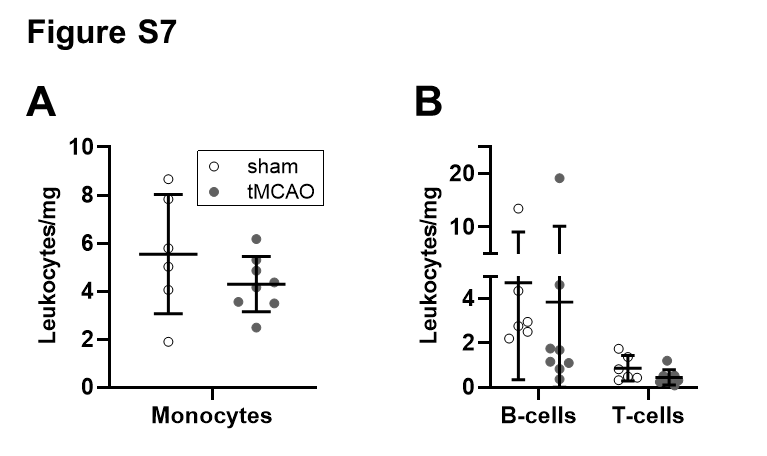
**

**Figure S7.** **Cardiac monocytes and lymphocytes do not differ after tMCAO**

Male mice (12-14 weeks old) received right-sided tMCAO (60 min) (filled circles) or sham (open circles). **A)** Monocytes, **B)** B- and T cells were assessed by flow cytometry.

**
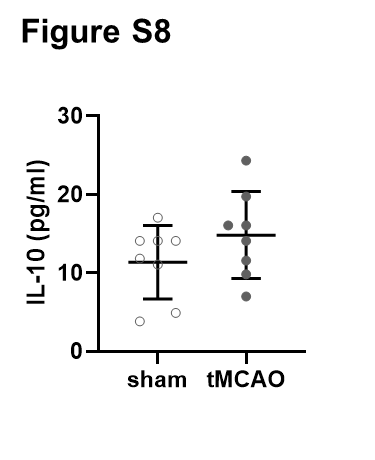
**

**Figure S8.** **Circulating levels of Interleukin-10 do not show significant changes in tMCAO mice as compared to sham mice**

Male mice (12-14 weeks old) received right-sided tMCAO (60 min) or sham operation and plasma cytokines were assessed by Bio-Plex assay 24 hours after intervention.

**
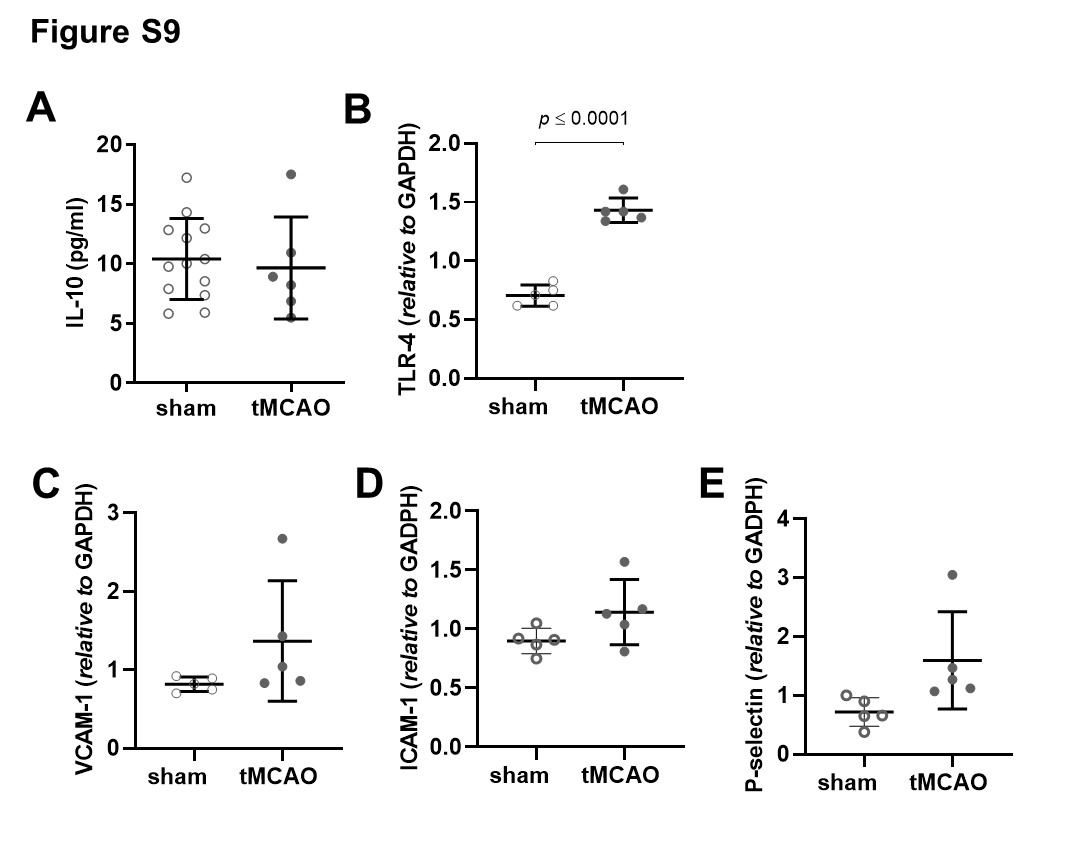
**

**Figure S9.** **Upregulation of Toll-like-receptor-4 and unchanged IL-10 expression in cardiac tissue of tMCAO mice compared to sham controls**

Male mice (12-14 weeks old) received right-sided tMCAO (60 min) or sham operation.

**A)** Interleukin (IL)-10 were assessed by Bio-Plex assay 24 hours after intervention. **B)** Toll-like-receptor (TLR)-4 and **C)** VCAM-1, **D)** ICAM-1 and **E)** P-selectin were analyzed by quantitative polymerase chain reaction.

**
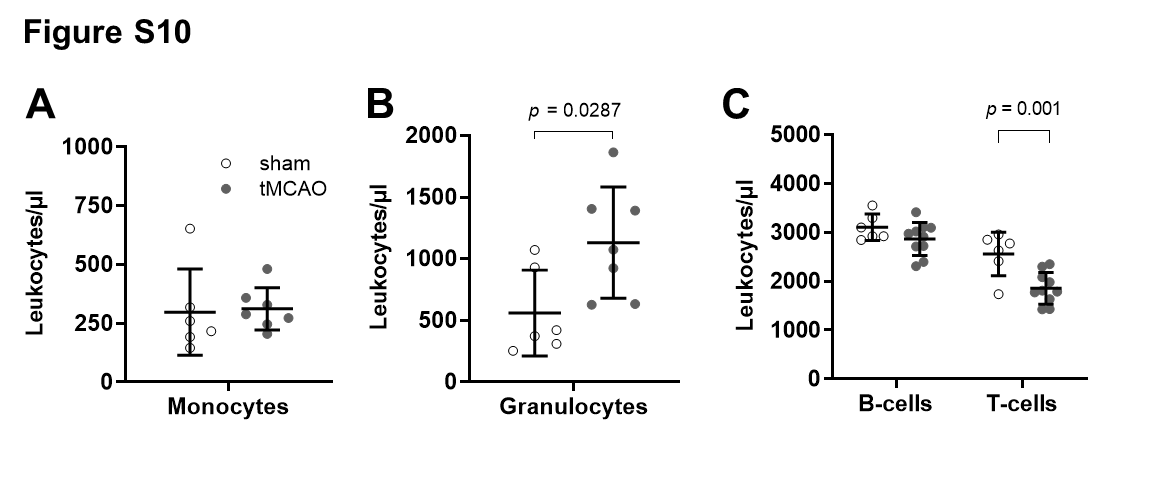
**

**Figure S10.** **Splenic granulocytes increased, while T-cells decreased following tMCAO**

Male mice (12-14 weeks old) received right-sided tMCAO (60 min) or sham operation and splenic leukocytes were assessed by flow cytrometry 24 hours after intervention.

**
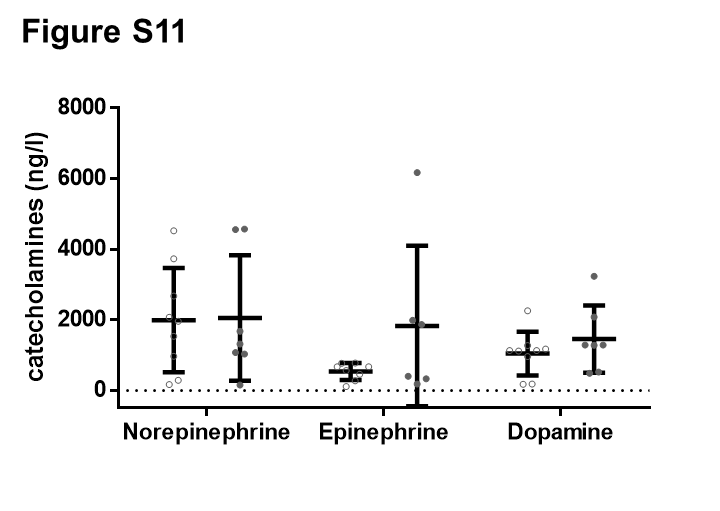
**

**Figure S11.** **Plasma catecholamines were increased only by tendency in tMCAO animals**

**A)** Male mice (12-14 weeks old) received right-sided tMCAO (60 min) or sham operation and plasma catecholamines were assessed by ELISA 24 hours after intervention.

**
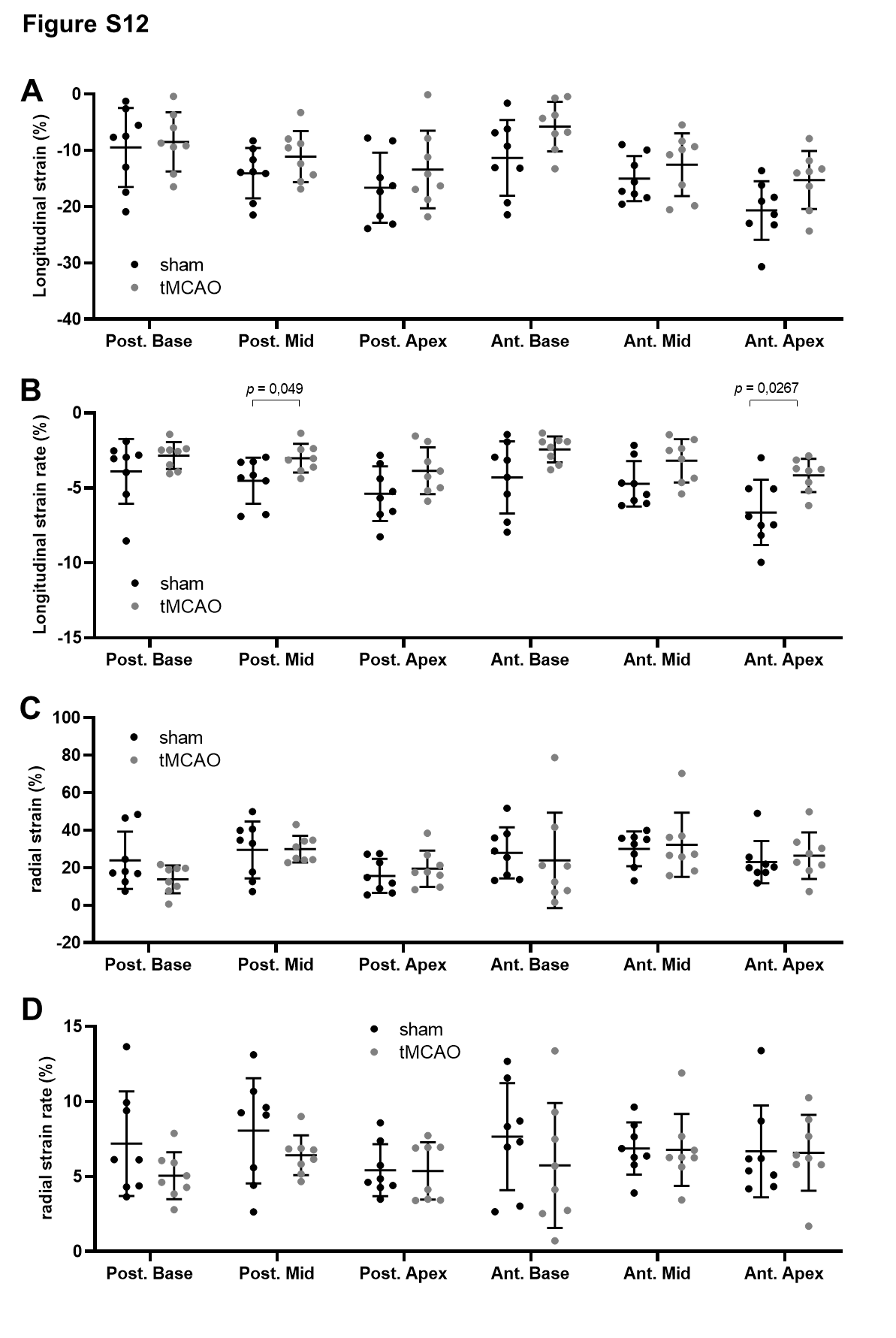
**

**Figure S12.** **Strain- and Strainrate analysis**

Male mice (12-14 weeks old) received right-sided tMCAO (60 min) or sham operation and cardiac function was assessed 24 h after surgery by echocardiography.

**A)** Longitudinal strain was calculated in six segments (anterior and posterior base, mid and apex).

**B)** Longitudinal strain rate was calculated in six segments (anterior and posterior base, mid and apex).

**C)** Radial strain was calculated in six segments (anterior and posterior base, mid and apex).

**D)** Radial strain rate was calculated in six segments (anterior and posterior base, mid and apex).

**
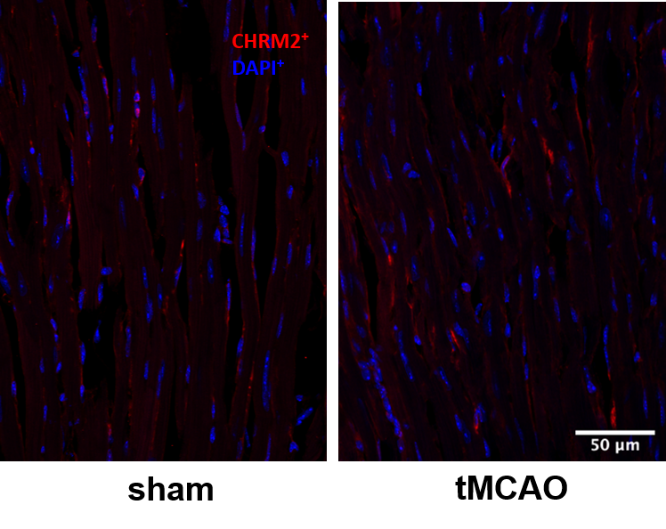
Figure S13**

**Figure S13 Determination of muscarinic acetylcholine receptor-2 density**

Male mice (12-14 weeks old) received right-sided tMCAO (60 min) or sham operation. Hearts (24h) were analyzed by immunefluorescence for expression of muscarinic acetylcholine receptor-2 (CHRM2). CHRM2 and DAPI areas were quantified in seven representative hearts sections.

**
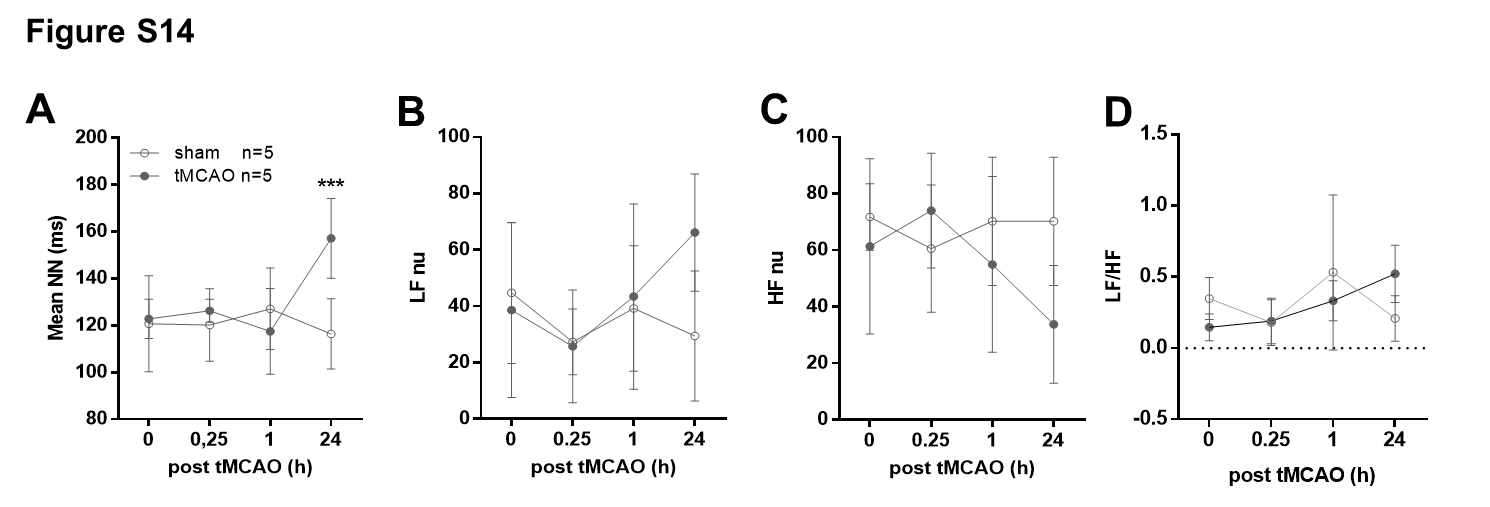
Figure S14 Heart rate variability (HRV) and LF/HF-ratio evaluation demonstrated an increase in tMCAO compared to sham**

Male mice (12-14 weeks old) received right-sided tMCAO (60 min) or sham operation. ECG-recordings were performed at baseline (0h), after 15 minutes (0.25h), after 1 hour (1h) and after 24 hours (24h).

1. HRV (Mean NN) was evaluated in tMCAO mice and sham mice at 4 timepoints.
2. Low frequency band (LF) was measured in tMCAO mice and sham mice at 4 timepoints.
3. High frequency band (HF) was measured in tMCAO mice and sham mice at 4 timepoints.
4. LF/HF-ratio was calculated for tMCAO mice and sham mice at 4 timepoints.

**Supplemental Tables:**

**Table T1** Bederson Score for neurological examination


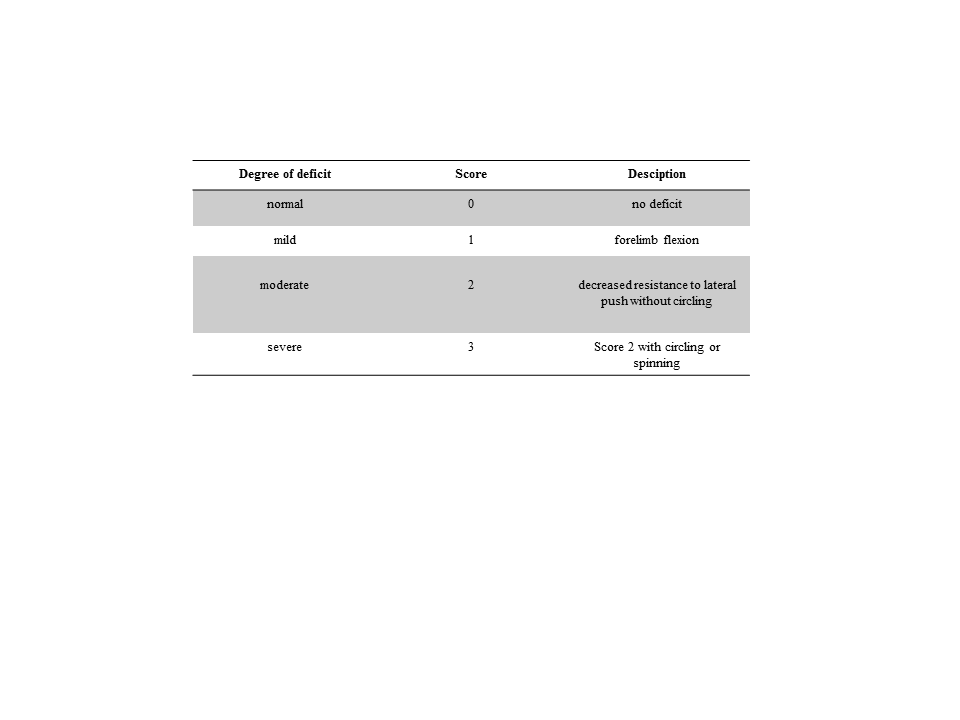


**Table T2** Preparation of flow buffer for flow cytometry

| **Buffer** | **Chemicals** | **Volume** | **Company** | **Country** |
| --- | --- | --- | --- | --- |
| **Flow buffer** | Phosphate buffered solution (PBS) | 500 ml | Sigma-Aldrich | Germany |
|  | Fetal calve serum | 2 % | Sigma-Aldrich | Germany |
|  | Ethylenediaminetetraacetic acid | 2 mM | Sigma-Aldrich | Germany |

**Table T3** Preparation of washing buffer for flow cytometry

| **Buffer** | **Chemicals** | **Volumes** | **Company** | **Country** |
| --- | --- | --- | --- | --- |
| **Washing buffer** | PBS | 1 liter | Sigma-Aldrich | Germany |
|  | Sodium bicarbonate | 4 mM | Sigma-Aldrich | Germany |
|  | HEPES buffer | 10 mM | Sigma-Aldrich | Germany |
|  | Diacetyl monoxim | 30 mM | Sigma-Aldrich | Germany |
|  | Glucose | 11 mM | Sigma-Aldrich | Germany |
|  | Egtazic acid | 0.3 mM | Roth | Germany |
|  | Sodium chloride | 6.6 mM | vwr | Germany |
|  | Potassium chloride | 0.22 mM | Merck | Germany |
|  | Magnesium chloride·H_2_O | 0.1 mM | Sigma-Aldrich | Germany |
|  | *fumigated in carbogen (95 % O_2_, 5 % CO_2_), pH 7.4* | | | |

| **Solution** | **Chemicals** | **Volumes** | **Company** | **Country** |
| --- | --- | --- | --- | --- |
| **Collagenase solution** | Collagenase NB8 | 1.2 mg | serva | USA |
|  | Hanks` balanced Salt solution | 20 % | gibco | USA |
|  | Washing buffer (see Table 2) | 80 % | see Table 2 | see Table 2 |

**Table T4** Preparation of collagenase solution for isolation of cardiac leukocytes
